# Phase recognition in SEM-EDX chemical maps using positive matrix factorization

**DOI:** 10.1016/j.mex.2023.102384

**Published:** 2023-09-26

**Authors:** Xiangrui Kong, Ivana Staničić, Viktor Andersson, Tobias Mattisson, Jan B.C. Pettersson

**Affiliations:** a Department of Chemistry and Molecular Biology, Atmospheric Science, University of Gothenburg, Gothenburg SE-412 96, Sweden; b Department of Space, Earth and Environment, Chalmers University of Technology, Gothenburg SE-412 96, Sweden

**Keywords:** PMF analysis of SEM-EDX images, PMF, SEM, EDX, Chemical looping, Non-negative matrix factorization

## Abstract

Images from scanning electron microscopy (SEM) coupled with energy-dispersive X-ray spectroscopy (EDX) are informative and useful to understand the chemical composition and mixing state of solid materials. Positive matrix factorization (PMF) is a multivariate factor analysis technique that has been used in many applications, and the method is here applied to identify factors that can describe common features between elemental SEM-EDX maps. The procedures of converting both graphics and digital images to PMF input files are introduced, and the PMF analysis is exemplified with an open-access PMF program. A case study of oxygen carrier materials from oxygen carrier aided combustion is presented, and the results show that PMF successfully groups elements into factors, and the maps of these factors are visualized. The produced images provide further information on ash interactions and composition of distinct chemical layers. The method can handle all types of chemical maps and the method is not limited solely to SEM-EDX although these images have been used as an example. The main characteristics of the method are:

• Adapting graphics and digital images ready for PMF analysis.
• Conversion between 1-D and 2-D datasets allows visualization of common chemical maps of elements grouped in factors.
• Handles all types of chemical mappings and large data sets.

## Graphical abstract

**Figure:**
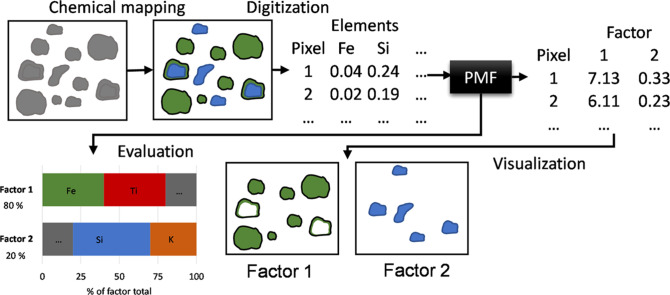

Specifications table

**Table:** 

Subject area	Chemical Engineering
More specific subject area	*Chemical looping, material analysis*
Name of your method	*PMF analysis of SEM-EDX images*
Name and reference of original method	*PMF (Paatero P,* et al.*, Environmetrics 1994; 5: 111–126.)*
Resource availability	*The analysis can be carried out in any PMF program, and the EPA PMF 5.0 used in the study is an open-source program.*

## Introduction

Scanning electron microscopy (SEM) serves as an analytical tool for studying topography, morphology, microstructures and chemical distribution of various sample types. Micrographs are obtained by rastering a focused electron beam across the surface, detecting secondary or backscattered electron signals. The incorporation of energy dispersive x-ray (EDX) analyzer allows for identification of elements and determining the quantitative composition. Consequently, each pixel in a micrograph is associated with an EDX spectrum containing both qualitative and quantitative data. However, despite the comprehensive nature of the EDX spectrum, it falls short of providing insights into the phases present within the sample. Nevertheless, there are several methods available that can extract useful information from the created images. A common approach involves correlating elements and identifying features by superimposing elemental maps based on designated hypotheses. Alternative post-processing methods have been reported for investigating chemical maps. For example, Germinario et al. [1] employed digital image analysis to treat X-ray maps, extracting relative abundances of distinct mineral phases. This specific approach entails pre-processing each elemental map and superimposing a selection of maps. The resulting superimposed maps are analyzed as multispectral images and various phases are identified based on the chemical composition and segmented according to manually defined training pixels. Another study by Allegretta et al. [2] employs a method wherein spectral information is processed by fitting a mathematical model, utilizing the EDX spectrum within each pixel. This technique is employed for the identification and segmentation of diverse phases, as demonstrated in the study of complex soil aggregates.

This paper introduces a novel method that employs positive matrix factorization (PMF) [3] for identifying and visualizing phases within chemical maps. In contrast to the previous methods, the following approach can be applied without pretreatment of the chemical maps. The inherent flexibility of PMF allows uncovering patterns and sources that may not be immediately apparent, making it particularly suitable for capturing intricate relationships within the data and thus phase analysis. PMF, also known as non-negative matrix factorization (NMF) [4], is a multivariate factor analysis technique that has been successfully used in many environmental and chemometric evaluation applications [5]. The function of the PMF is to identify factors that can describe common features between targeted datasets, *e.g.,* elemental maps obtained by SEM-EDX. However, the current PMF tools require the input datasets as vectors, *i.e.,* one quantity as a function of time or other types of sequence, but the 2-D image matrices are not readily useable for PMF analysis. In this paper, we illustrate an approach to utilize the PMF method on 2-D SEM-EDX images, which will result in clustering results that show the correlations between elements and their spatial distributions. Moreover, this approach is not limited to SEM-EDX images but can be generally applied to 2-D matrix datasets and can easily be extended to 3-D datasets.

### Sample information and preparation

To illustrate the strength of the proposed method, micrographs of a bed material sampled after combustion of biomass will be used as an example. The sample consists of particles in the range of 100–200 µm and the particles have been used in a 115 MW_th_ CFB boiler for Oxygen Carrier Aided Combustion (OCAC) of biomass where ilmenite, an iron-titanium mineral, was utilized as the bed material [6,7]. This type of bed material is also referred to as oxygen carrier (OC). Since the boiler in question usually operates using silica sand, there are inevitably silica sand particles found in the sample. When utilizing OCs for combustion of solid fuels the particles will inevitably interact with inorganic species present in the ash. The most commonly investigated interactions between a fluidized bed material and biomass ash involve silicon-containing bed material. However, interactions between ash and an oxygen carrying bed material, such as ilmenite, have also been studied but to a significantly lesser extent [8]. One common approach is to study the chemical mappings of the particle surfaces and cross-sections obtained by SEM/EDX. Electron micrographs and chemical maps provide information regarding the sample characteristics, such as the particle type (*e.g.*, ilmenite or silica sand), ash layer composition, particle shapes, structure (*e.g.*, porosity), *etc*.

**Fig. 1:**
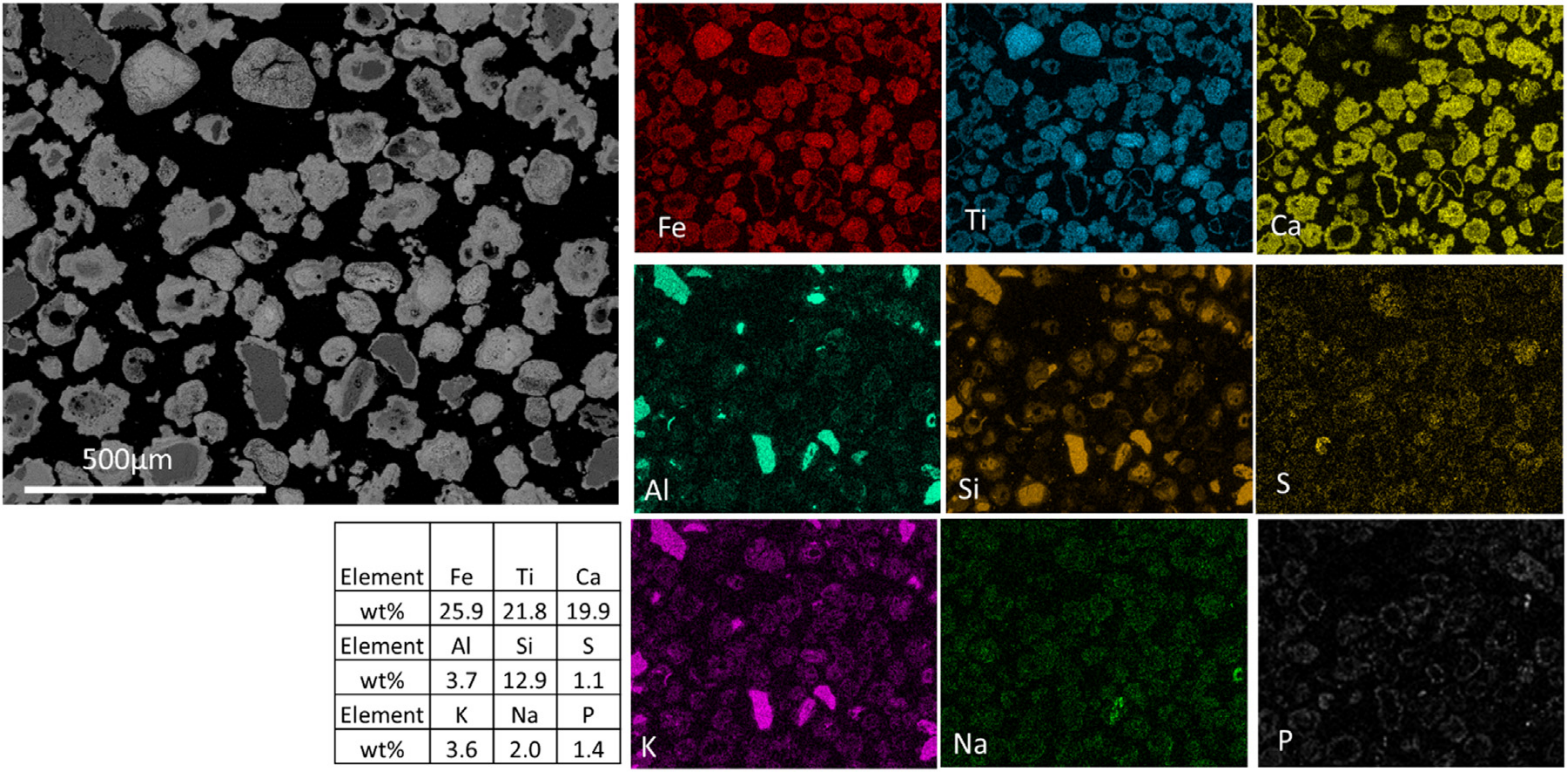
Micrograph of the cross-section of an ilmenite sample. Chemical map of the major components of ilmenite (Fe and Ti), major ash elements (Ca and Si) along with Al, P, and the alkali species (K and Na). The total content in weight percent is presented in the table (excluding C and O).

To study the particle cross-section, particles were immobilized in a mixture of epoxy and hardener. When cured, the samples were polished stepwise to obtain a flat surface and expose the particle cross-section. This way, knowledge of the distributions of inorganic ash species within the particles and ash layers can be obtained. Fig. 1 shows the cross-sections (particles are cut and their cores are visible) of heterogeneously mixed particles of different chemical compositions. The system used for imaging and chemical mapping was the Quanta 200 ESEM FEG from FEI equipped with an Oxford EDX system. The maps of common elements are shown in individual panels. The heterogeneity in the sample is reflected in two aspects:

(1) A fraction of the particles shows distinguishable elemental compositions. Ilmenite particles are distinguished by high iron and titanium concentrations while silica particles have higher aluminum, alkali, and silicon concentrations.
(2) Some elements are enriched on surfaces whereas some are only present in the cores. For example, the elements Fe-Ti-Ca seem to cover some particles with the K-Al-Si elements in the core.

In general, these correlations between elements indicate the chemical species, as well as kinetic behavior of different elements when comparing samples that have experienced different processes [9]. This is a common message expected from the SEM-EDX analysis. However, with the PMF approach it is possible to, for example, identify the fraction of ilmenite in a mixture with silica sand, the fraction of an element associated with a specific phase and/or different ash layers within the sample.

**Fig. 2:**
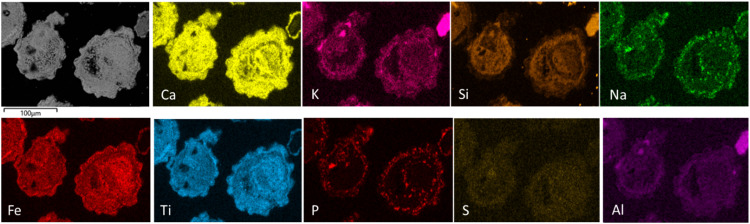
Cross-section micrograph of an ilmenite particle after Oxygen Carrier Aided Combustion in a 115 MW_th_ biomass-fired boiler. The mappings of the major ilmenite components (Fe and Ti) along with the ash elements Ca, K, P, Si, Al, Na, and S.

Fig. 2 presents the chemical mapping of the cross-section of two ilmenite particles used in OCAC of biomass. A selection of ash components (Ca, K, Si, Na, P, S, and Al) is presented in the figure, and visible primarily in the outer layer of the particle. Ca is also detected across the particle cross-section together with Fe and Ti. The correlation between elements can be studied by point analyses, line scans, and chemical mappings. But from these chemical mappings, it is difficult to see the correlation between different elements. It will be shown that with the PMF analysis, a statistical approach is used to divide the elements into different factors that represents possible phases within the particle.

## Method

The method presented here centers around utilizing images of acceptable quality, with contrast being the primary determinant rather than absolute brightness or color. Pre-processing the utilized chemical maps is not required. However, it's important to highlight that changes in brightness, without compromising contrast, does not impact the results. On the other hand, elevating brightness to the maximum level can result in information loss and subsequently alter the outcome of the PMF analysis. In essence, the alteration occurs due to reduced contrast. To clarify further, if brightness is adjusted while retaining contrast, it poses no issue but if brightness is increased to its maximum extent, diminishing the pixel differences and degrading picture quality, the PMF result might be affected. This notion aligns with the concept of normalization, as changing brightness equates to altering absolute numbers. Nonetheless, as long as the relative pixel differences persist, maintaining picture contrast, the images remain suitable for analysis. Therefore, the method retains its robustness by effectively working with images containing distinguishable objects, even in cases where these objects are challenging to observe visually.

Prior to the PMF-analysis, the chemical maps need to be digitized and converted to vectors. To utilize the 2-D matrices, the following procedure needs to be applied, which arranges the matrices into vectors. Also, the targeted images can be in either graphics or digital format. In the former case, the images need to be digitized before use, and in the latter case, the digital image files can be analyzed directly. The digitization can be carried out in several ways, and here follows an example approach using MatLab to digitize and arrange a graphics file, *e.g.*, in *png* format (named “S1_Al.png”).

**Table:** 

###################################	
S1_Al = imread('S1_Al.png');	% Read images, to *unit8* format with three color dimensions (RGB)
S1_Al_1 = im2double(S1_Al(:,:,1));	% Convert image to single color dimension in double precision
S1_Al_2 = im2double(S1_Al(:,:,2));	% Convert image to single color dimension in double precision
S1_Al_3 = im2double(S1_Al(:,:,3));	% Convert image to single color dimension in double precision
S1_Al_1 = reshape(S1_Al_1,1,[]);	% Convert 2D matrix into vector
S1_Al_1 = transpose(S1_Al_1);	% Transpose a row vector to a column vector
S1_Al_2 = reshape(S1_Al_2,1,[]);	% Convert 2D matrix into vector
S1_Al_2 = transpose(S1_Al_2);	% Transpose a row vector to a column vector
S1_Al_3 = reshape(S1_Al_3,1,[]);	% Convert 2D matrix into vector
S1_Al_3 = transpose(S1_Al_3);	% Transpose a row vector to a column vector
writematrix(S1_Al_1)	% Write the vector to a file
writematrix(S1_Al_2)	% Write the vector to a file
writematrix(S1_Al_3)	% Write the vector to a file
###################################	

The last three rows generate three vectors (S1_A1_1, S1_A1_2, S1_A1_3) of RGB codes (red, green, blue) of the image. These vectors are converted from 2-D image matrices, as the PMF analysis requires a dataset in vector format rather than matrix format. These vectors can be considered 1-D images. However, although one dimension is reduced, these vectors can be reversely converted back to matrices, as the conversion rule is known.

The color tone of images can lead to certain vectors being blank or, occasionally, two vectors having equal strength (like blue and red vectors forming purple images). To simplify without compromising quality, the preferred approach is to select the vector with the strongest signals. In the case of purple images, either a red or blue vector works equally well. For black and white images, any vector is suitable. Importantly, this step can be omitted for digitally formated files.

The vectors of interesting elements are compiled into a single file and used as a PMF input file, which can be saved as .txt, .csv, or .xls. Table 1 shows an example file. The columns are the elements, and the rows are the positions of the 1-D images. Normalization can be optionally applied to each column, *i.e.*, the maximum of each column is unity. The uncertainty is estimated based on the instrument characteristics, *e.g.*, 1 % is used in this study and is also saved in .xls format.

**Table 1:** Example of a .xls PMF input file, where only the first 17 rows are listed, and the remaining are omitted for clarity.

Al	Mg	Ni	S	C	O	P	Si	Ca
0.00E+00	8.61E-02	0.00E+00	0.00E+00	0.00E+00	0.00E+00	9.91E-02	0.00E+00	0.00E+00
4.10E-03	4.10E-03	1.39E-01	0.00E+00	4.00E-02	8.06E-03	9.43E-03	1.31E-02	1.37E-02
4.10E-03	1.23E-02	3.33E-02	0.00E+00	5.14E-02	8.06E-03	4.72E-03	0.00E+00	0.00E+00
4.10E-03	4.10E-03	1.22E-01	4.44E-02	5.71E-02	3.23E-02	0.00E+00	0.00E+00	0.00E+00
4.10E-03	8.20E-03	2.78E-02	5.56E-03	7.43E-02	8.06E-03	4.72E-03	0.00E+00	0.00E+00
4.10E-03	8.20E-03	1.50E-01	3.33E-02	4.57E-02	1.61E-02	4.72E-03	0.00E+00	0.00E+00
4.10E-03	8.20E-03	9.44E-02	4.44E-02	7.43E-02	1.61E-02	4.72E-03	6.54E-03	0.00E+00
4.10E-03	1.64E-02	5.00E-02	1.11E-02	9.71E-02	1.61E-02	9.43E-03	0.00E+00	0.00E+00
4.10E-03	4.10E-03	3.89E-02	5.56E-02	7.43E-02	8.06E-03	4.72E-03	1.31E-02	0.00E+00
4.10E-03	4.10E-03	8.89E-02	5.56E-03	5.71E-03	1.61E-02	9.43E-03	6.54E-03	0.00E+00
4.10E-03	2.05E-02	1.22E-01	1.11E-02	6.86E-02	1.61E-02	0.00E+00	6.54E-03	0.00E+00
4.10E-03	2.05E-02	1.44E-01	0.00E+00	8.57E-02	8.06E-03	4.72E-03	6.54E-03	0.00E+00
4.10E-03	1.23E-02	8.89E-02	5.56E-03	1.71E-02	1.61E-02	0.00E+00	0.00E+00	0.00E+00
4.10E-03	2.46E-02	1.06E-01	0.00E+00	7.43E-02	1.61E-02	4.72E-03	1.31E-02	0.00E+00
4.10E-03	1.64E-02	6.67E-02	5.56E-03	6.86E-02	3.23E-02	9.43E-03	0.00E+00	0.00E+00
4.10E-03	0.00E+00	7.78E-02	0.00E+00	2.86E-02	8.06E-03	0.00E+00	6.54E-03	0.00E+00
4.10E-03	8.20E-03	6.67E-02	0.00E+00	5.71E-03	0.00E+00	0.00E+00	0.00E+00	0.00E+00

Several PMF programs are available, and an open-source EPA PMF program is used here to illustrate the procedure [10]. The program, along with the user guide, can be downloaded from the following Ref. [11]. After loading the concentration and uncertainty files to the program, a number of runs are chosen (default is 20) along with the number of factors. When the analysis is done, a few output files are generated, and the “Profile” and “Contributions” sheets are the key results, which will be used for illustration in later parts of this paper.

## Case study

The data in Fig. 2 is converted to a PMF input file according to the procedure presented above to produce vector matrices that are used in EPA PMF 5.0 developed by the US Environmental Protection Agency [10]. In case an image is too large, the image can be resized before converting to a 1-D vector. Note that this is a limitation of the EPA PMF 5.0 software and not the PMF method. A separate Excel file is created stating the uncertainty for each element, which was set to 1 %. After loading the concentration and uncertainty files in PMF-EPA under the tab “Model Data”, an output folder and prefix are specified. Next, something that is possible in the program but has not been done here is to exclude data. This can for example be done to remove outliers. After the data has been specified under “Model Data”, the settings are specified in the tab “Base Model”. The number of runs for this example was 20 (default) and the number of factors (4) was specified. As with other forms of factor analysis, the number of factors needs to be specified. This number is chosen based on knowledge about the sample characteristics. Due to the complex distribution of the elements, different number of factors were investigated, and it was concluded that a total of four factors provide results with physical meaning. Examples with three and five factors are illustrated in the results. After choosing the number of factors the program was run and a few output files were generated. The file named XXX_base.xlsx contains the main result. In this Excel file two sheets, “Profile” and “Contributions”, contain the key results.

## Results and discussion

**Fig. 3:**
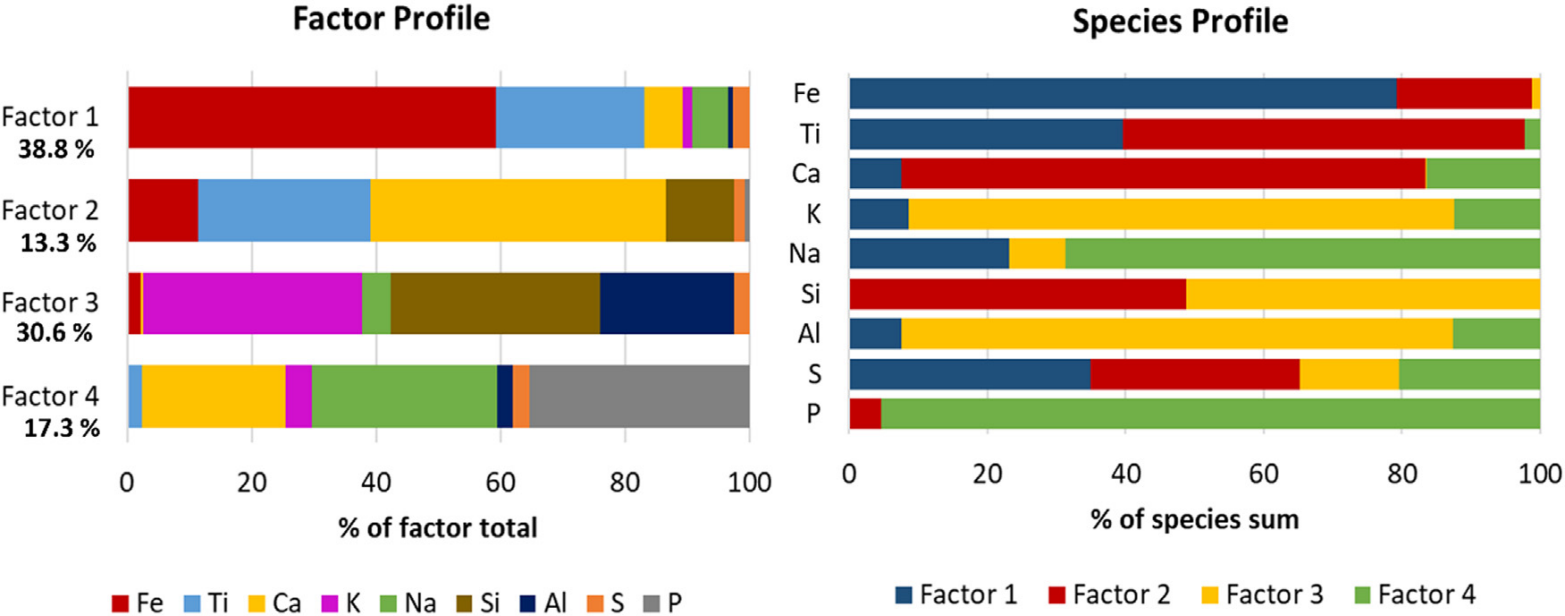
Factor profiles (left) and species profile (right) after PMF-analysis of Fig. 2. The factor profile shows what species the factors are comprised of and the species profile shows how each species is distributed amongst the different factors.

In the “Profile”-sheet different tables are available with information on the species and factors. Data from the tables are used to create the stacked column figures presented in Fig. 3, which show how the factors are distributed among the species where the left is normalized with respect to the species (Species profile) and the right with respect to the factors (Factor profile). The composition of each factor is completely different.

From the factor profile, one can observe that Ti is associated with both Factor 1 and 2 while the species profile shows that a higher amount of Ti is associated with Factor 2. From these profiles, the difference between K and Na is evident. The major amount of K is associated with aluminosilicates (Factor 3) while Na on the other hand coexists with P (Factor 4).

**Fig. 4:**
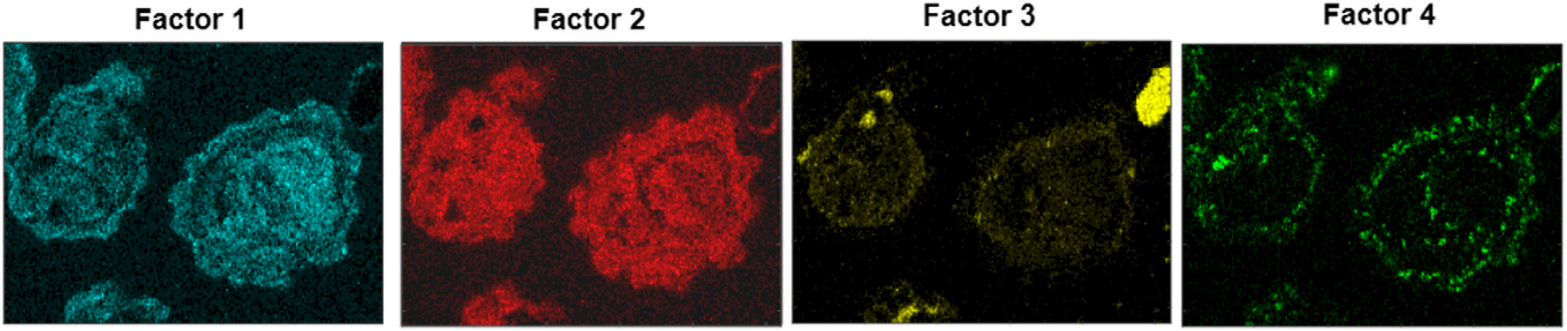
Images representing the four-factor profiles after reconverting the one-dimensional results from the “Contributions”-sheet back to images.

In the “Contributions” sheet a table can be found. In the table, each row represents a position in the image, and each column contains the corresponding factor values. These vectors were reversely converted back to matrices in Matlab. Images with the factor distributions are presented in Fig. 4. From the figure, different ash layers and interactions can be observed and understood. The results may be used to identify phases based on the chemical composition of the factors and phase separation based on the plotted factors. For example, it is known that a separation of Fe from Ti occurs when ilmenite is used in consecutive redox cycles [12].

The enhanced iron concentration in the outer layer (Factor 1) and the titanium-rich core (Factor 2) is visible in Fig. 4. From the crystallographic results reported in literature [13] the major phases are pseudobrookite (Fe_2_TiO_5_), perovskite (CaTiO_3_) and feldspar (KAlSi_3_O_8_) which are well in aligned with factors 1–3 respectively.

However, the PMF analysis provides additional information showing that a phosphate phase is also present. The factor profiles also provide an indication of the distribution between the phases. The visualization of Factor 4 in Fig. 4shows that the combined Ca and Na phosphate is primarily concentrated in the outer layers of the particles. From the factor profile in Fig. 3, it is shown that the titanium-rich core (Factor 2) also includes the elements Ca and Si while the outer ash layer (Factor 1) correlates with K, Na, Ca, and S. This information provides furter insights in the composition of each phases. Furthermore, it is likely that Factor 1, which contains a small amount of K and Ti, partly contains potassium titanate which has previously been observed in OCAC operation with ilmenite [14]. Factor 2 is present across the particles, as seen in Fig. 4, which indicates diffusion of for example Ca into the particle core. The major K-containing factor is Factor 3 and likely in the form of potassium aluminosilicates (potassium feldspar). It is primarily present in some individual particles as seen in Fig. 4.

**Fig. 5:**
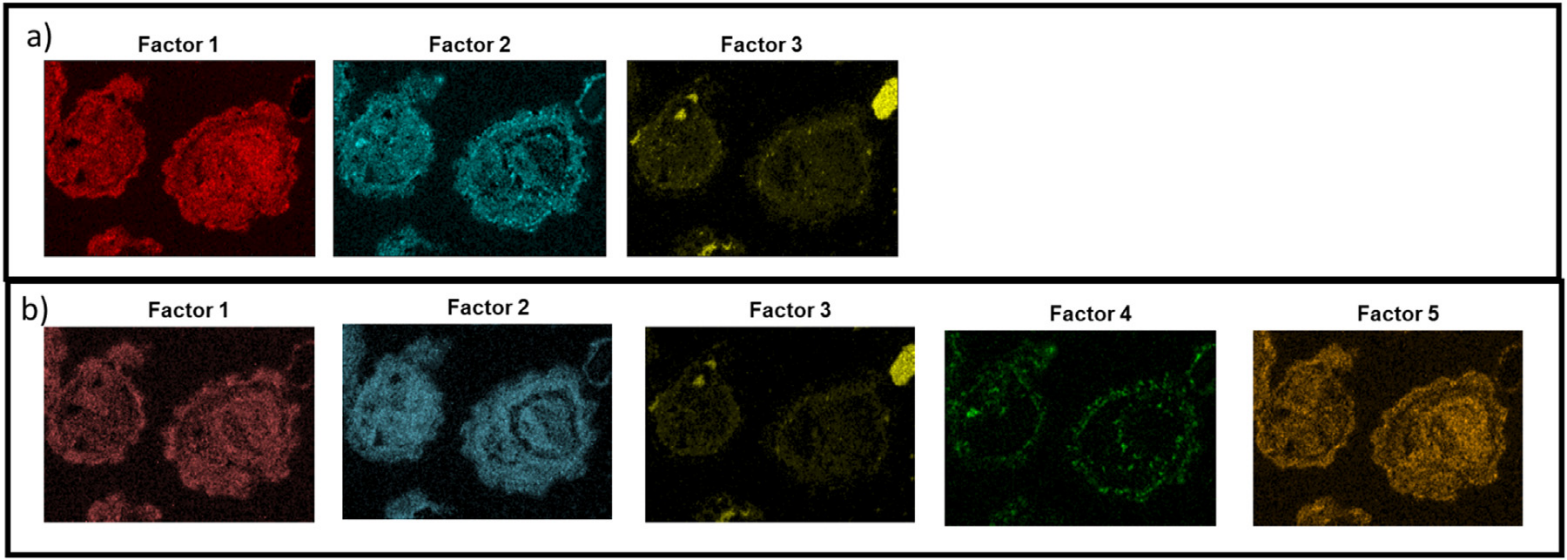
Images of a three-factor profile (panel a) and five-factor profile (panel b) after reconverting the one-dimensional results from the “Contributions”-sheet back to images.

Although the method introduced in this study eliminate the need for phase characterization, it remains crucial to specify the number of factors, and thereby the number of phases. This value varies for each unique sample. Unfortunately, the selection of factor count significantly impacts the outcomes. An excessive number of factors essentially produce the elemental maps, whereas too few factors could potentially lead to the oversight of important features. To illustrate the latter scenario, Fig. 5 demonstrates three and five-factor profiles in panels a and b respectively. Using three factors (Fig. 5a) shows that the analysis fails to discern the phosphate phase, resulting in data loss. Using more than four factors, see example in Fig. 5b, produces factor profiles where one element dominates and does not provide new information. As such, every sample necessitates careful consideration when determining the optimal factor count to ensure meaningful results.

## Conclusions

This paper presents a method to analyze micrographs obtained from spectroscopy by grouping elements into factors and revisualizing the results. The method introduced here correlates elements based on their spatial distributions, using the PMF method. It can handle all types of chemical maps and the method is not limited to SEM-EDX data although these images have been used as an example. Furthermore, this method can handle large data sets. When the method is applied to material samples from chemical looping processes, the produced factor images provide information on co-existence of elements by visualizing distinct chemical layers and phases.

## CRediT authorship contribution statement

**Xiangrui Kong:** Conceptualization, Formal analysis, Methodology, Writing – original draft. **Ivana Staničić:** Investigation, Formal analysis, Methodology, Software, Writing – original draft. **Viktor Andersson:** Resources, Writing – review & editing. **Tobias Mattisson:** Supervision, Writing – review & editing. **Jan B.C. Pettersson:** Supervision, Writing – review & editing.

## Declaration of Competing Interest

The authors declare that they have no known competing financial interests or personal relationships that could have appeared to influence the work reported in this paper.

## Data Availability

Data will be made available on request.
